# Identification and Expression Profiling of the Auxin Response Factors in *Dendrobium officinale* under Abiotic Stresses

**DOI:** 10.3390/ijms18050927

**Published:** 2017-05-04

**Authors:** Zhehao Chen, Ye Yuan, Di Fu, Chenjia Shen, Yanjun Yang

**Affiliations:** 1 College of Life and Environmental Sciences, Hangzhou Normal University, Hangzhou 310036, China; zhchen@hznu.edu.cn (Z.C.); y_yuan1130@126.com (Y.Y.); fudiclh@163.com (D.F.); shencj@hznu.edu.cn (C.S.); 2 Zhejiang Provincial Key Laboratory for Genetic Improvement and Quality Control of Medicinal Plants, Hangzhou Normal University, Hangzhou 310036, China

**Keywords:** abiotic stress, auxin, *ARF*, *Dendrobium officinale*, transcriptional activator

## Abstract

Auxin response factor (ARF) proteins play roles in plant responses to diverse environmental stresses by binding specifically to the auxin response element in the promoters of target genes. Using our latest public *Dendrobium* transcriptomes, a comprehensive characterization and analysis of 14 *DnARF* genes were performed. Three selected *DnARFs*, including *DnARF1*, *DnARF4*, and *DnARF6*, were confirmed to be nuclear proteins according to their transient expression in epidermal cells of *Nicotiana benthamiana* leaves. Furthermore, the transcription activation abilities of *DnARF1*, *DnARF4*, and *DnARF6* were tested in a yeast system. Our data showed that *DnARF6* is a transcriptional activator in *Dendrobium officinale*. To uncover the basic information of *DnARF* gene responses to abiotic stresses, we analyzed their expression patterns under various hormones and abiotic treatments. Based on our data, several hormones and significant stress responsive *DnARF* genes have been identified. Since auxin and *ARF* genes have been identified in many plant species, our data is imperative to reveal the function of *ARF* mediated auxin signaling in the adaptation to the challenging *Dendrobium* environment.

## 1. Introduction

Phytohormones play pivotal roles in the various aspects of plant growth and development, including embryogenesis, organogenesis, root architecture, flower and fruit development, tissue and organ patterning, vascular development, and secondary metabolism [1,2,3,4]. In plants, auxin signaling is transmitted by transcriptional regulation of some auxin early responsive gene families, such as Auxin/Indole-3-acetic acid (Aux/IAA), Gretchen Hagen3 (GH3) and Small Auxin Up RNA (SAUR) [5]. Auxin response factor (ARF) proteins, an essential component of the auxin signaling pathway, regulate the expression levels of auxin response genes by targeting the auxin response elements (AuxREs) on their promoters [6]. A typical ARF protein consists of three conserved domains: a plant specific B3-like DNA-binding domain (DBD) at the N-terminus, an activation domain (AD) or repression domain (RD) in the middle region, and a conserved C-terminal dimerization domain (CTD) [7].

In the past years, several *ARF* gene mutants were screened to investigate the genes’ genetic functions. In *Arabidopsis*, an *ARF2* loss-of-function mutant shows severe leaf senescence and floral organ abscission [8]. Another mutant, *AtARF3*, displays deviant floral meristem and reproductive organs [9]. The abnormal formation of vascular strands and the embryo axis are observed in the mutant *AtARF5* [10]. A mutation in the *AtARF7* gene blocks the hypocotyl response to blue light and auxin stimuli, and shows differential growth in aerial tissues [11]. A T-DNA insertion mutant in *ATARF8* was reported to control reproductive organ development by uncoupling fertilization and pollination from fruit development [12]. Furthermore, studies of double mutants of the *AtARF* genes provide insight into their overlapping functions. The *AtARF2* gene regulates leaf senescence and floral organ abscission independently of the ethylene and cytokinin response pathways, and the *AtARF1*/*ATARF2* double mutant enhanced many *AtARF2* phenotypes [13]. The *AtARF6*/*AtARF8* double mutant flowers are presented as infertile closed buds with short petals, short stamen filaments, undehiscent anthers and immature gynoecia, indicating that the *AtARF6* and *AtARF8* gene dosage quantitatively affects the timing of flower maturation [14]. Another double mutant, *AtARF7*/*AtARF19*, has a strong auxin-related phenotype, resulting in severely impaired lateral root formation and abnormal gravitropism in both hypocotyls and roots [15]. This suggests that *AtARF7* and *AtARF19* play essential roles in auxin mediated plant development by regulating both unique and partially overlapping sets of target genes [16]. The genome-wide identification and expression analysis of *ARF* genes in tomato also indicated that *ARF* genes may play diverse roles during the reproductive developmental stages in Solanaceae in general [17].

Recent studies have uncovered the involvement of *ARFs* in responses to environmental stress. In rice, *OsARF16* is required for iron and phosphate starvation responses [18,19]. Another *ARF*, *OsARF12*, is also involved in phosphate homeostasis. Expression profiling using qRT-PCR and microarray data revealed many water stress-responsive *ARF* genes in soybeans [20]. In tea plants, the expression of several *CsARFs* significantly changed under salt and dehydration stresses [21]. Expression responses of ARFs to abiotic stress also has been identified in banana [22].

*Dendrobium*, the second largest genus in the Orchidaceae, consists of more than 1000 species with high ornamental and medicinal values [23,24]. Stems of some *Dendrobium* species, such as *Dendrobium officinale*, contain compounds that exhibit antioxidant and antitumor activities, resulting in their high commercial values as traditional medicines [25,26]. Both the transcriptome and genome of *D. officinale* have been sequenced, allowing for the isolation and identification of auxin-related genes [27]. Since first cloned from *Arabidopsis* (*AtARF1*), 22 members in tomato (*Solanum lycopersicon*), 31 members in maize (*Zea mays* L.), 15 members in cucumber (*Cucumis sativus*), 39 members in poplar (*Populus trichocarpa*), 25 members in rice (*Oryza sativa* L.), 24 members in *Medicago* (*Medicago truncatula*), 19 members in sweet orange (*Citrus sinensis*), and 51 members in soybean (*Glycine max* L.) have already been identified [6,17,18,28,29,30,31,32]. In our study, we identified *14 ARF family* genes in *D. officinale*, and analyzed their expression patterns under different hormones and abiotic stresses. Because auxin and *ARF* genes have been identified in many plant species, it is important to reveal the functions of ARF mediated auxin signaling in *Dendrobium*’s adaptation to challenging environments.

## 2. Results

### 2.1. Isolation of 14 DnARF Genes from the D. officinale Plants

Based on our previous transcriptome data, more than 30 candidate *ARF* genes were identified in the *D. officinale* plants [27]. The sequences that shared a high open reading frame (ORF) identity (>99%) with other candidate *DnARF* genes were excluded from our study (data not shown). In total, 14 *DnARF* genes with full-length ORFs were identified and named according to the nomenclature. The information for these *DnARF* genes, such as Unigene IDs, gene names, MR locations, ORF lengths, and parameters for the deduced polypeptides, are listed in Table 1. The sizes of the deduced DnARF proteins varied markedly from 522 amino acids (DnARF17) to 981 amino acids (DnARF19b). The corresponding molecular masses varied from 57.58 kDa to 109.66 kDa, and the predicted isoelectric points varied from 5.08 (DnARF19b) to 8.40 (DnARF17).

### 2.2. Isolation of 14 DnARF Genes from the D. officinale Plants

**Figure 1:**
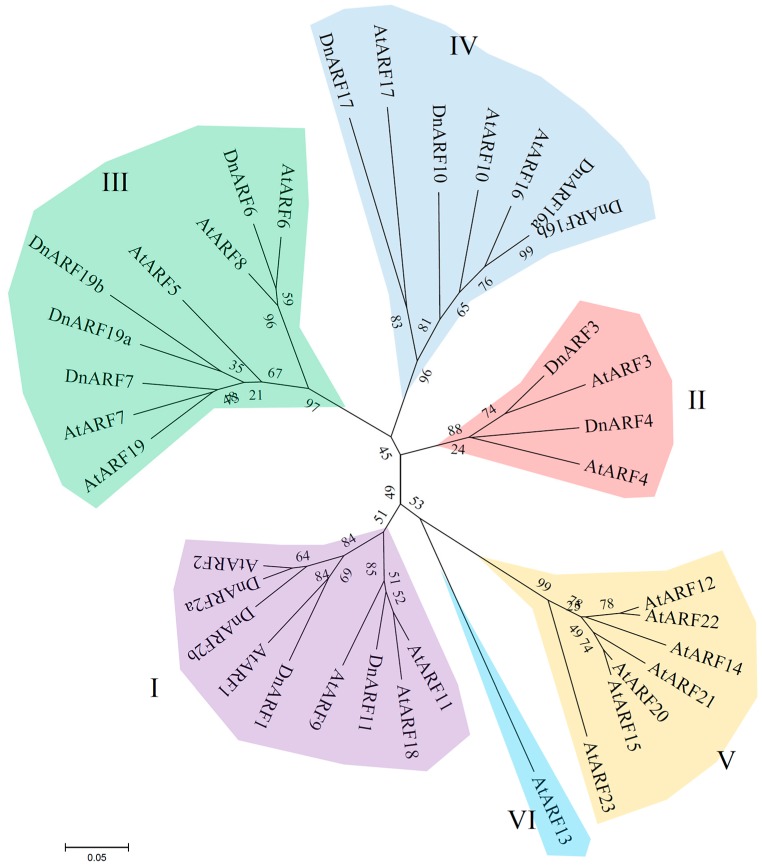
Phylogenetic relationships of *Arabidopsis* and *D. officinale* ARF proteins. An unrooted phylogenetic tree was constructed using MEGA 5.1 (The Biodesign Institute, Tempe, AZ, USA) by N–J method. Bootstrap values are presented for all branches. All *ARF* family genes were grouped into six subgroups named from I to VI. Different subfamilies were showed by different colorized shading.

**Figure 2:**
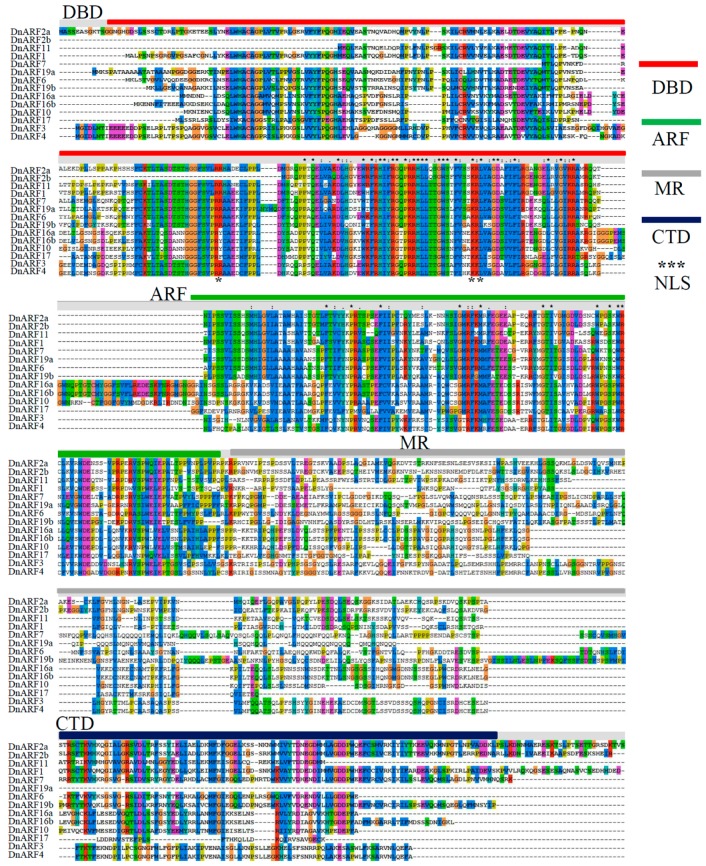
Protein sequences alignment and domain analysis of DnARF family proteins. Alignment of DnARF proteins obtained with the ClustalW program under default parameters. Multiple alignments of the domains DBD, ARF, MR, and CTD of the DnARF proteins were showed by different color lines. DBD: B3 DNA-binding domain; ARF: AUX_RESP domain; MR: middle region; CTD: C-terminal dimerization domain; NLS: nuclear localization signals. Colorized shading indicates identical and conversed amino acid residues, respectively. Two NLSs were marked by black asterisks.

**Figure 3:**
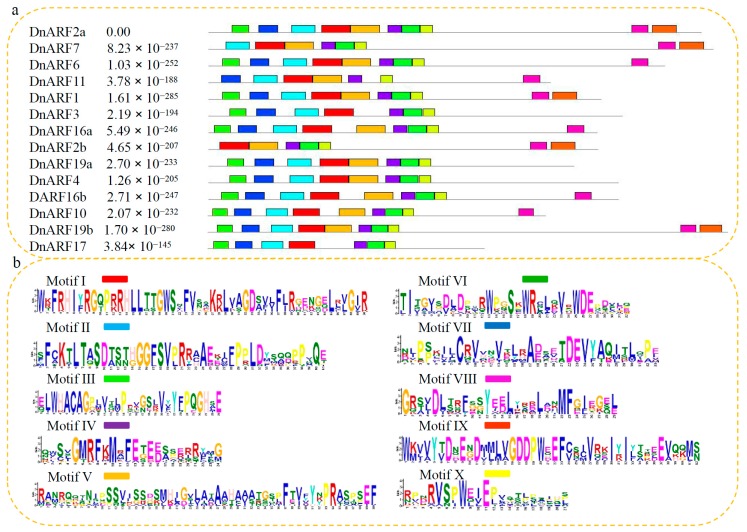
Analysis of motif distribution in DnARF proteins. Analysis of motif distribution in DnARF proteins. (**a**) Ten classical motifs in DnARF proteins were analyzed by MEME (Multiple Em for Motif Elicitation) online tool. The width of each motif ranged from six to 50 amino acids. Different color blocks represent different motifs. (**b**) Analysis of specific amino acid conservation in each motif. The height of each character represents different conservative degrees.

A phylogenetic tree was built to explore the evolutionary relationship of ARFs between *D. officinale* and *Arabidopsis*. The phylogenetic distribution revealed that ARF proteins can be grouped into six subgroups, I, II, III, IV, V, and VI (Figure 1). The 14 DnARFs were not evenly distributed among the six different subgroups, and the subgroups V and VI were AtARF specific subgroups. The deduced polypeptides of the 14 DnARFs were used in a multiple sequence alignment, and their similarities were shown in Figure 2. Most of the DnARF proteins contained three conserved domains, DBD, ARF and CTD. There is a variable MR between the ARF and CTD. The results were consistent with the multiple alignments. Furthermore, two typical putative nuclear localization signals (NLS) were detected in all of the DnARF proteins: a short lysine/arginine amino acid sequence with a bipartite NLS structure was located between domains I and II, and a monopartite NLS of simian virus 40, which contains two stretches of lysine/arginine residues, was located at the end of domain II (Figure 2). The domains were next analyzed on the MEME website. Ten conserved motifs were identified and mapped to the DnARF protein sequences (Figure 3a). Motifs II, III, and IIV corresponded to the DBD; Motifs I and V corresponded to the ARF domain; and Motifs XIII and IX corresponded to the CTD. Of the 14 identified DnARFs, only DnARF4, 17 and 19a lacked the CTD. In addition, DBD was missing from two DnARFs, including DnARF2b and *7*. All DnARFs contained a conserved ARF domain. Moreover, identical and conversed amino acid residues were indicated by different colorized shading (Figure 3b).

**Figure 4:**
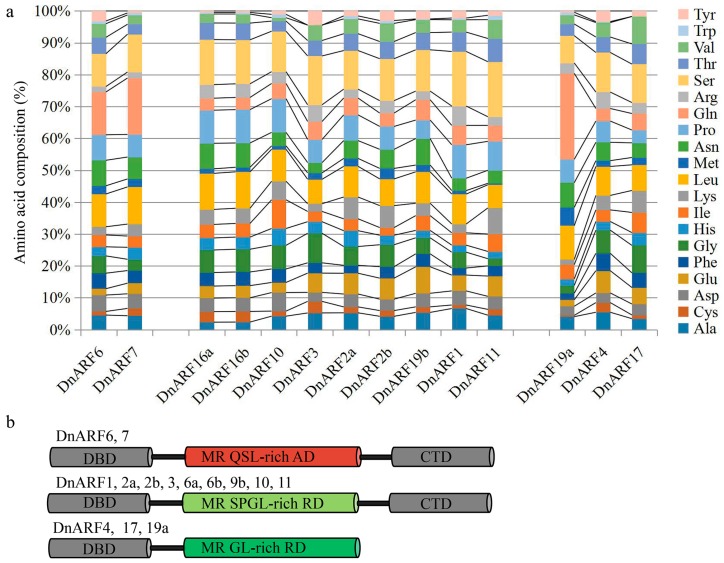
Analysis and classification of ARF family in *D. officinale*. (**a**) Amino acid compositions of the MR domains in various DnARF proteins. Different colors indicated different types of amino acids. (**b**) Classification of DnARF proteins based on their amino acid preferences and domain structures. DBD: B3 DNA-binding domain; MR: middle region; CTD: C-terminal dimerization domain; Q: glutamine; S: serine; L: leucine; G: glycine; P: proline; AD: activation domain; RD: repression domain.

The amino acid compositions of the MRs and the classifications of the DnARFs were shown in Figure 4. The domain positions in the 14 DnARF proteins were listed in Table 1. The data showed that most of the DnARFs contained conserved DBDs, and variable MR and CTDs [7,28]. The 14 DnARFs can be divided into three classes based on the MR amino acid composition and the presence of CTDs. The DnARF family includes only two putative transcriptional activators, DnARF6 and *7*, which contain an MR enriched in glutamine, serine, and leucine. This type of MR functions as an active domain in *Arabidopsis* or rice ARFs [7,33,34]. Most DnARFs, including DnARF1, 2a, 2b, 3, 6a, 6b, 9b, 10 and 11, are putative transcriptional repressors with MRs enriched in serine, leucine, proline, and glycine, similar to the *Arabidopsis* repressors [7,33]. DnARF4, 17and 19a do not contain a CTD.

### 2.3. Subcellular Localization and Transcriptional Activation of Three Selected DnARFs

**Figure 5:**
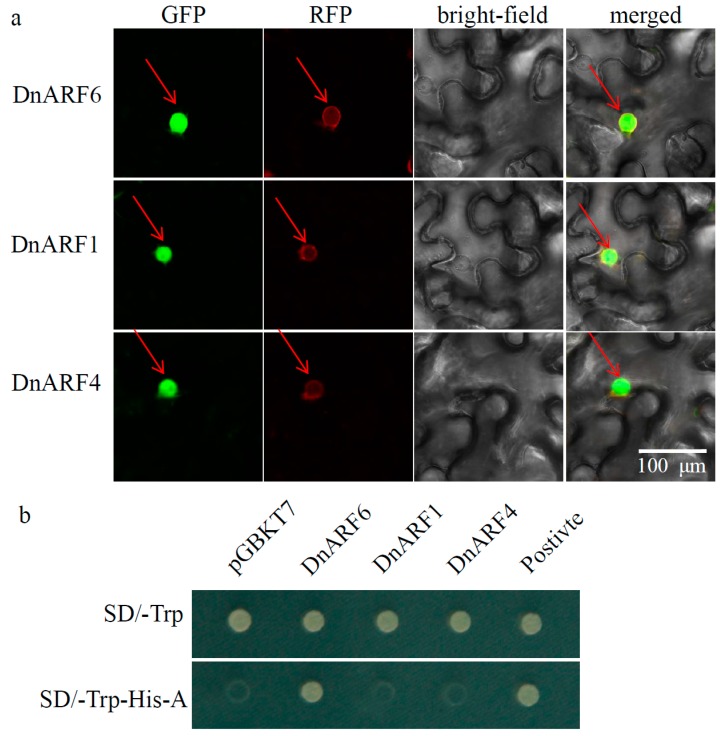
Subcellular localization and transcriptional activation of three selected DnARFs. (**a**) *DnARF* gene-GFP fusion constructs transiently expressed in tobacco epidermis cells. Localization of DnARF6, DnARF1, and DnARF4 fusion protein. Left to right: green fluorescence, red fluorescence, bright-field and merged. (**b**) Transcriptional activities of DnARF6, DnARF1, and DnARF4 were tested by the yeast system. The growth of transformed yeast strain AH109 with constructs under SD/-Trp and SD/-Trp-His-A nutrition-deficient medium. BD refers to the pGBKT7 vector, which serves as the negative control.

Based on the results of the amino acid composition analysis, a transcription activator, DnARF6, a transcription repressor, DnARF1, and the CTD-truncated DnARF4 were selected for transcriptional activation tests. Firstly, the full-length ORFs of these three genes were fused in-frame to the N terminus of the GFP coding region. According to previous reports in other plant species, *ARFs* are nuclear-localized proteins [34]. We observed the transient expression of DnARF6, DnARF1, and DnARF4 in the epidermal cells of *N. benthamiana* leaves, confirming that they are nuclear proteins (Figure 5a).

To analyze the transcription activation capabilities of DnARF6, DnARF1, and DnARF4, autonomous activation tests were performed in the yeast system. On the SD medium lacking tryptophan, yeast strains containing one of the four constructs: empty BD, BD: DnARF6, BD: DnARF1, and BD: DnARF4, as well as a positive control, could grow well, indicating successful transformations. On the triple nutrient deficient SD medium, the strains containing the DnARF6 and the positive control could grow well; however, the strain with the empty BD, DnARF1, and DnARF4 did not survive (Figure 5b). Our data confirmed that DnARF6 may function as a transcriptional activator in *D. officinale*.

### 2.4. Expression Patterns for DnARF Genes in Different D. officinale Organs

**Figure 6:**
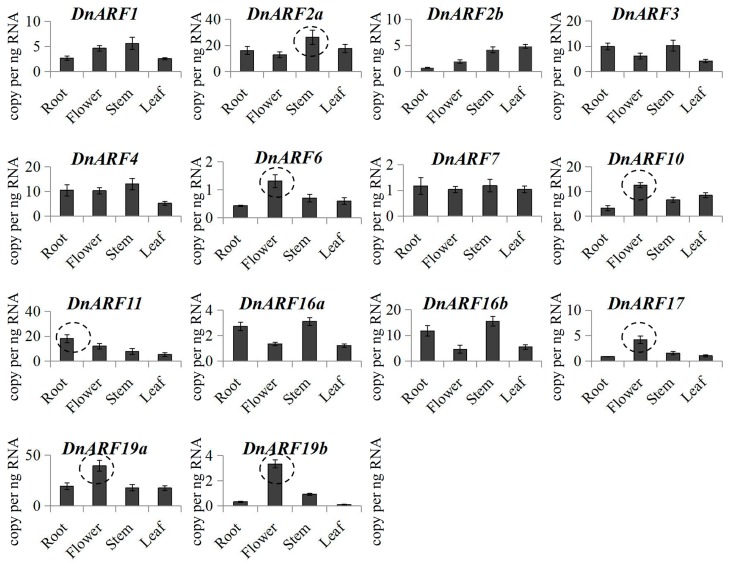
Organ-specific expression of *DnARF* family genes. Expression patterns of *DnARF* genes in four organs, including root, flower, stem, and leaf. The highest expression accumulation in organs was marked by dash line circles.

In our study, qRT-PCR was used to examine the spatial specificity of the expression patterns of each *DnARF* gene in different *D. officinale* organs. The transcript accumulations of these *DnARF* genes could be detected in at least one organ, and most of these genes expressed ubiquitously in all of the organs (Figure 6). The transcript levels of DnARF2b, DnARF17 and DnARF19b were hardly detectable in roots, suggesting their limited roles in root growth and development. DnARF6, DnARF10, DnARF17, DnARF19a and DnARF19b expressed much higher in the flowers than in other organs, and DnARF2a displayed the highest expression accumulation in the stems. DnARF11 had root-specific expression, indicating its role in root system establishment.

### 2.5. Expression of DnARF Genes in Response to Various Hormone Treatments

**Figure 7:**
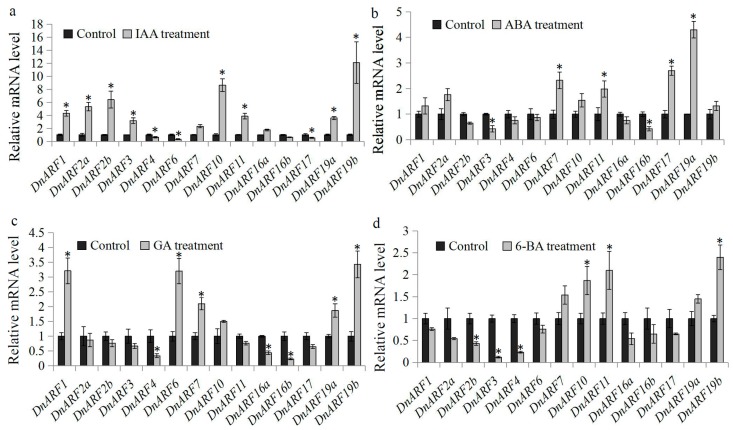
Expression analysis of *DnARF* genes under various hormone treatments. Total RNA was extracted from the seedlings of *D. officinale* for basal expression. The relative expression levels of 14 *DnARF* genes under (**a**) IAA, (**b**) ABA, (**c**) GA, and (**d**) 6-BA treatments. Significant differences in expression of DnARF genes between control and hormone treatments were indicated by ‘*’.

Previous study has revealed the involvement of hormones in plant responses to abiotic stresses [35]. In our study, the expression levels of the *DnARF* family genes in *D. officinale* seedlings were tested by qRT-PCR under Indole-3-acetic acid (IAA), abscisic acid (ABA), gibberellins (GA) and 6-Benzylaminopurine (6-BA) treatments, respectively. The expression levels of *DnARF1*, *DnARF2a*, *DnARF2b*, *DnARF3*, *DnARF10*, *DnARF11*, *DnARF9a*, and *DnARF19b* were significantly induced by the IAA treatment, and *DnARF6* and *DnARF17* were significantly reduced by the IAA treatment (Figure 7a). Under the ABA treatment, the expression levels of *DnARF7*, *DnARF11*, *DnARF17*, and *DnARF19a* were significantly up-regulated and the expression levels of *DnARF3* and *DnARF16b* were significantly down-regulated (Figure 7b). Under the GA treatment, the expression levels of *DnARF1*, *DnARF6*, *DnARF7*, *DnARF19a*, and *DnARF19b* were significantly up-regulated and the expression levels of *DnARF4*, *DnARF16a*, and *DnARF16a* were significantly down-regulated (Figure 7c). Under the 6-BA treatment, the expression levels of *DnARF10*, *DnARF11*, and *DnARF19b* were significantly induced and the expression levels of *DnARF2a*, *DnARF3*, and *DnARF4* were significantly reduced (Figure 7d). The results suggested that the expression levels of some *DnARF* genes are responsive to these selected hormones.

### 2.6. Expression of DnARF Genes in Response to Various Abiotic Stress Treatments

Abiotic stresses, including high salinity, dehydration, and extreme temperatures, are frequently experienced by plants under natural conditions [36,37]. Auxin controlled gene transcriptional regulation is an essential process required for plants to survive and adapt to adverse environmental challenges [21]. In our study, the expression patterns of *DnARF* genes under NaCl, PEG, and low (4 °C) and high temperature (30 °C) treatments were analyzed to investigate their potential roles in *D. officinale* responses to various abiotic stresses.

**Figure 8:**
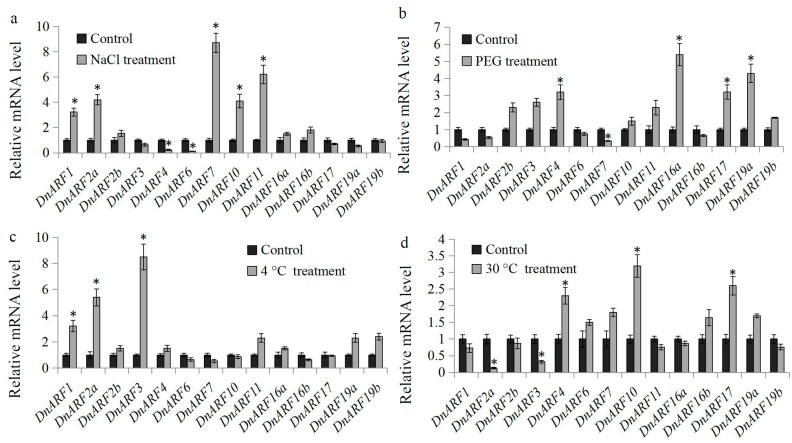
Expression analysis of *DnARF* genes under various abiotic treatments. Total RNA was extracted from the seedlings of *D. officinale* for basal expression. The relative expression levels of 14 *DnARF* genes under (**a**) NaCl, (**b**) PEG, (**c**) 4 °C, and (**d**) 30 °C treatments. Significant differences in expression of *DnARF* genes between control and abiotic treatments were indicated by “*”.

Under the NaCl treatment, five *DnARF* genes, *DnARF1*, *DnARF2a*, *DnARF7*, *DnARF10*, and *DnARF11*, were significantly up-regulated, and only two genes, *DnARF4* and *DnARF6*, were significantly down-regulated (Figure 8a). The expression levels of *DnARF4*, *DnARF16a*, *DnARF17*, and *DnARF19a* were significantly induced and the expression level of *DnARF7* was significantly reduced by the PEG treatment (Figure 8b). Under the cold treatment, *DnARF1*, *DnARF2a*, and *DnARF3* were significantly induced, and no significantly reduced *DnARF* genes were found (Figure 8c). The high temperature treatment significantly increased the expression levels of *DnARF4*, *DnARF10*, and *DnARF17* and decreased the expression levels of *DnARF2a* and *DnARF3* (Figure 8d). These results indicated that some *DnARF* genes are transcriptional responsive to abiotic stresses.

## 3. Discussion

The phytohormone auxin is involved in regulating many aspects of plant growth and development [38,39,40,41]. ARF proteins form large and multigenic families in various plant species [18,19]. The isolation of *ARF* gene families in plants aids in the understanding of their functions in growth and developmental processes [34,42]. The availability of sequence information in the public databases and transcriptomes in our laboratory allowed us to identify ARF family members in *D. officinale*. In this study, 14 *Dendrobium* ARFs were identified and their expression patterns were analyzed. Because the sequence lengths were limited to those of the transcriptomics unigenes, only 14 *ARF* genes with full-length coding sequences were identified in *D. officinale*, which is less than the numbers of *ARF* family genes in other higher plants [6,28,34]. Considering the large size of the *D. officinale* genome, we believe that more *ARFs* exist in *D. officinale* [43]. For example, members of the subgroups V and VI were not identified in *D. officinale*, probably due to the incomplete sequence information from the transcriptome data (Figure 1).

A large number of *ARFs* have been widely reported in many species. Our evolutionary analysis of *DnARF* genes with those from *A. thaliana* elucidated the conservation of *ARF* genes between *D. officinale* and the model plant. The subgroups I, II, III, and IV were conserved in the *ARF* genes of both *D. officinale* and *Arabidopsis*. All of the *DnARF* genes were distributed in four subgroups, I to IV which are homologous to *AtARF1/2*, *AtARF3/4*, *AtARF5/6* and *AtARF10/16*, respectively (Figure 1). The evolutionary relationships of these genes between *D. officinale* and *Arabidopsis* suggested the putative biological functions of these newly identified *ARFs* [6,31,32].

In this study, all of the 14 DnARF proteins contained a B3-like DBD and an ARF domain (Figure 2). Three DnARF proteins; DnARF4, 17 and 19a, lacked a CTD, an important domain responsible for the interactions between *ARFs* and Auxin/IAAs (Figure 4b). The percentage of the CTD truncated DnARFs was 21.4%, which was similar to that in sweet orange (21.1%), tomato (28.6%), rice (25.0%) and *Brassica rapa* (22.6%), and lower than that in papaya (36.4%) and *Medicago* (58.3%) [5,34,44,45]. This suggested that the gene expression regulation in *D. officinale* occurred in an auxin independent manner [30,46]. Many studies have verified that the transcriptional activities of ARFs depend on the amino acid composition of the MRs [28,34]. In our study, only two DnARF proteins; DnARF6 and DnARF7, were predicted as transcriptional activators, suggesting roles in the activation of the downstream target genes (Figure 4b). Furthermore, their transcriptional activation capabilities were confirmed by the yeast two-hybrid system (Figure 5b).

Hormones are involved in plant responses to changing environmental stimuli and stresses by affecting the expression levels of many *ARF* genes in different plant species [20,21]. However, the evaluation of various commercial hybrids and varieties of *Dendrobium* for suitability to the shifting surroundings is very limited. In our study, a systematic expression profile of the *D. officinale ARF* family genes under several hormones and different abiotic stresses was created.

The responsiveness to auxin treatments is a major feature of *ARF* genes in plants [28,47]. In *D. officinale*, 11 of the 14 *DnARF* genes showed significant changes in expression levels under IAA treatments compared with the control (Figure 7a). Our results are consistent with several previous reports on maize, cucumber and *Medicago* [6,48,49]. In addition to auxin, the role of the hormone cross-talk in response to abiotic stress has been uncovered [50]. In *Arabidopsis*, the stimulation of ABA signaling during seed dormancy is controlled by inducing *ARF* mediated ABI3 activation, which suggests a coordinating network of auxin and ABA signals [51]. For example, auxin acts upstream of ABI3 by recruiting the *ARF10* and *ARF16* to control the expression of ABI3 during seed germination [51]. Moreover, the transcriptional regulation of GA metabolism-related genes is also controlled by auxin signaling [52]. In tomato, *SlARF7* mediates cross-talk between auxin and GA signaling during fruit set and development [53]. The differential expression of *DnARF* genes under ABA and GA treatments suggested the presence of hormone cross-talk in *D. officinale* (Figure 7b,c). Recently, some abiotic stress-responsive *ARF* genes have been reported in banana [22]. In sorghum, the expression levels of the *SbARF10*, *16*, and *21* genes are significantly increased, over 10-fold, when subjected to a dehydration treatment [54]. In soybean seedlings, more than half of the *ARF* genes (33 in 51 *GmARFs*) are dehydration responsive in shoots and/or roots [20]. To our knowledge, there are few studies on the molecular mechanisms the environmental tolerance of *D. officinale* plants. Therefore, our data provided us many excellent candidates for further studies.

## 4. Materials and Methods

### 4.1. Plant Material, Growth Conditions, and Treatments

*D. officinale* seedlings were incubated in a greenhouse located at Hangzhou Normal University, Hangzhou, China. The three year old adult plants were transferred into independent pots and grown at a temperature of 25 ± 1 °C with a light/dark cycle of 12/12 h and 60–70% relative humidity [55]. The leaf, stem, root, and flower samples were collected from the *D. officinale* plants during the flowering stage. There were three biological replicates for each organ. For hormone treatments, three year old plants were cultured in liquid half-strength Murashige and Skoog (MS) medium (Sigma-Aldrich, St. Louis, MO, USA), with 2% sucrose, at pH 5.4 as controls. Plants were soaked in liquid half-strength MS supplemented with 10 μM IAA, 100 μM abscisic acid (ABA), 100 μM gibberellic acid (GA), or 10 μM 6-benzylaminopurine (6-BA) for 3 days. In the salt stress experiment, *D. officinale* plants were soaked in half-strength MS containing 150 mM NaCl for 3 days. In the dehydration stress experiment, *D. officinale* plants were treated with 20% polyethylene glycol (PEG) 6000 for 3 days. For the treatment, *D. officinale* plants were kept in greenhouse at a temperature of 4 ± 1 °C for 3 days. Untreated seedlings were used as controls.

### 4.2. Isolation and Identification of ARF Genes in D. officinale Plants

The partial sequences of the *ARF* family genes were screened from four transcriptome data sets that were previously published [56]. All of the sequencing data sets are available at NCBI Sequence Read Archive (http://www.ncbi.nlm.nih.gov/sra/) under accession IDs SRR2014227, SRR2014230, SRR2014236, SRR2014246, SRR2014297, SRR2014325, SRR2014396, and SRR2014476. All of the target unigenes were identified using the BLASTX algorithm, and sequences sharing more than 70% identity were assembled using SeqMan software in the Lasergene package. The 14 assembled sequences were analyzed and identified as full length cDNA sequences of *ARF* genes in *D. officinale*. Furthermore, the hidden Markov model (HMM) profiles of the *ARF* protein family (Pfam 02309: AUX/IAA family; Pfam 06507: Auxin response factor (AUX_RESP), Pfam 02362: B3 DNA binding domain (B3) were employed to identify the *ARF* genes from *D. officinale*. The sequences were sorted as unique sequences for a further protein domain search using InterProScan Sequence Search (http://www.ebi.ac.uk/Tools/pfa/iprscan/).

### 4.3. Sequence Analysis, Phylogenetic Tree Building, and Prediction of Amino Acid Contents

A multiple sequence alignment was performed for the DnARF protein sequences using ClustalW with the default parameters. Four classical domains, I, II, III, and IV, were identified in most of the DnARF proteins based on the alignments results. A phylogenetic tree was constructed with the 14 aligned DnARF protein sequences and 23 AtARF protein sequences using MEGA5.1 (http://www.megasoftware.net/mega5/mega.html) employing the neighbor joining method. Bootstrap values were calculated from 1000 iterations. The Multiple Expectation Maximization for Motif Elicitation (MEME) web server (http://meme.nbcr.net/meme/cgi-bin/meme.cgi) was used to analyze motif distributions. Information on the *ARF* genes in *Arabidopsis* is listed in Table S1. The software MEGA 5.1 was also used to calculate the amino acid contents of the middle region (MR) domains in DnARF proteins. The classifications of DnARF proteins were based on the particular amino acid contents of the MRs. The activator domain, having a C-terminal domain (CTD), is glutamine/serine/leucine rich in the MR. The repressor domain having a CTD, is serine/proline/glycine/leucine rich in the MR, but the MR is glycine/leucine rich if there is no CTD.

### 4.4. RNA Isolation and Quantitative RT-PCR

Total RNA from different samples was extracted using a Plant RNeasy Mini kit (Qiagen, Hilden, Germany) according to the manufacturer’s instructions. The gene DnActin (comp205612_c0) was used as an internal standard to calculate relative fold differences based on the comparative cycle threshold (2^−ΔΔ*C*t^) values. The procedure of qRT-PCR was as follows: one μL of a 1/10 dilution of cDNA in ddH_2_O was add to five μL of 2× SYB Green buffer, 0.1 μM of each primer and ddH_2_O was then added to a final volume of 10 μL. The PCR conditions were 95 °C for 10 min, 40 cycles of 95 °C for 15 s and 60 °C for 60 s. All of the primer sequences are listed in Table S2. A histogram was constructed using the average values to visualize tissue specific expression levels.

Absolute quantification was used to calculate the tissue specific expression. The limit of detection and amplification efficiency of the qRT-PCR was carried out using 10-fold serial dilution of cDNA isolated from root sample, which was used to create a standard curve. Based on the standard curves, the slopes and correlation coefficients were used to calculate the PCR efficiency for each primer pairs. A formula: E = POWER (10, 1/slope) − 1 was used to calculate the PCR efficiency. Then, one μL cDNA (30 ng/μL) from different experiment samples were used as temples for qRT-PCR analysis. Based on the standard curve and PCR efficiency, threshold (2^−ΔΔ*C*t^) values were converted to copy per ng RNA.

### 4.5. Subcellular Localization Analysis

Full-length coding regions of two *DnARF* gene sequences were cloned into the vector pH7FWG2.0 to generate expression constructs. An artificial green fluorescent protein (GFP), fused in-frame to the C terminus of each DnARF protein, was placed under the control of a cauliflower mosaic virus 35S promoter. These constructs were transiently expressed in tobacco (*Nicotiana benthamiana*) epidermal cells using *Agrobacterium* mediated transformations. The fluorescence of the fusion protein constructs was detected using a confocal microscope LSM710 (Carl Zeiss, Oberkochen, Germany, http://corporate.zeiss.com/) [57].

### 4.6. Analysis of Transcriptional Activation

The Matchmaker yeast two hybrid system (Clontech, Mountain View, CA, USA) was used to detect the transcriptional activation of *DnARF6* and *DnARF7*. The deduced amino acid sequences of *DnARF6* and *DnARF7* were cloned and inserted into pGBKT7 in-frame fused with the GAL4 DBD to generate expression constructs. These constructs were transformed into yeast strain AH109 and selected on the minimal synthetic dextrose (SD) medium/-Trp and SD/-Trp-His-A to examine the reporter gene expression according to the Clontech Yeast Protocol Handbook. The interaction between the pGBKT7-p53 and pGADT7-SV40 large T-antigen was used as a positive control, and the empty pGBKT7 vector was used as a negative control.

### 4.7. Statistical Analysis

Differences between values were calculated using a one way analysis of variance with a Student’s *t*-test at a significance level of 0.05 in Excel software (Microsoft, Seattle, WA, USA). All of the expression analyses were performed for five biological repeats, and the values shown in the figures represent the average values of five repeats. The data are expressed as the means and standard deviations (mean ± SD).

## 5. Conclusions

In conclusion, we have collected comprehensive information, such as basic parameters, conserved domains, the amino acid compositions, subcellular localizations, transcriptional activations, and expression patterns in different organs and under different abiotic treatments, on 14 identified *DnARF* genes in *D. officinale*. The responsiveness of the *DnARF* genes to various hormones and stresses suggests that *DnARFs* are involved in the *D. officinale* plants’ tolerance to abiotic stresses.

## Figures and Tables

**Table 1:** The *ARF* family in *Dendrobium officinale*.

Gene	Locus ID	ORF (bp)	MR Locations	Deduced Polypeptide		
				Length (aa)	Mol wt (Da)	pI
*DnARF1*	comp169620_c0_seq10	2016	358–539	672	74,848.32	5.93
*DnARF2a*	comp173652_c0_seq13	2529	387–709	843	93,698.06	6.84
*DnARF2b*	comp164610_c0_seq15	1998	203–536	666	75,067.37	8.13
*DnARF3*	comp168166_c0_seq7	2124	380–628	708	77,816.9	6.49
*DnARF4*	comp163222_c1_seq4	2103	373–end	701	77,883.19	6.97
*DnARF6*	comp171031_c0_seq23	2340	361–711	780	87,556.94	6.39
*DnARF7*	comp173511_c0_seq20	2589	275–756	863	97,659.3	6.71
*DnARF10*	comp160144_c2_seq3	1914	383–572	638	71,309.5	6.74
*DnARF11*	comp170249_c2_seq3	1755	307–524	585	65,625.36	6.91
*DnARF16a*	comp165418_c0_seq20	1995	387–599	665	73,640.69	7.54
*DnARF16b*	comp160625_c0_seq3	2103	401–612	701	77,632.32	7.83
*DnARF17*	comp134032_c0_seq1	1566	349–end	522	57,580.99	8.40
*DnARF19a*	comp163679_c1_seq3	1875	381–end	625	70,303.81	8.09
*DnARF19b*	comp150031_c0_seq2	2943	360–78	981	109,549.1	5.08

ORF: open reading frame; MR: middle region; Mol wt: molecular weight; pI: isoelectric point.

## Supplementary Materials

Supplementary materials can be found at www.mdpi.com/1422-0067/18/5/927/s1.

## Abbreviations

*ARF*	Auxin response factor
Aux/IAA	Auxin/Indole-3-acetic acid
GH3	Gretchen Hagen3
SAUR	Small Auxin Up RNA
AuxREs	auxin response elements
DBD	DNA-binding domain
AD	activation domain
RD	repression domain
CTD	C-terminal dimerization domain
MS	Murashige and Skoog
ABA	abscisic acid
6-BA	6-benzylaminopurine
GA	gibberellic acid
PEG	polyethylene glycol
HMM	hidden Markov model
MEME	Multiple Expectation Maximization for Motif Elicitation
GFP	green fluorescent protein
ORF	open reading frame
