# Supplementary material for: Identification and Expression Profiling of the Auxin Response Factors in Dendrobium officinale under Abiotic Stresses
Source: Int J Mol Sci. 2017 May 4;18(5):927. doi: 10.3390/ijms18050927 (PMC5454840; doi:10.3390/ijms18050927)
Supplement: Supplementary file 1 [file ijms-18-00927-s001.pdf]

Table S1 The information of *ARF* family gene in *Arabidopsis*

Species<br>Arabidopsis	ID	gene name
	AtARF1	AT1G59750.1
	AtARF2	AT5G62000.1
	AtARF3	AT2G33860.1
	AtARF4	AT5G60450.1
	AtARF5	AT1G19850.1
	AtARF6	AT1G30330.2
	AtARF7	AT5G20730.1
	AtARF8	AT5G37020.1
	AtARF9	AT4G23980.1
	AtARF10	AT2G28350.1
	AtARF11	AT2G46530.3
	AtARF12	AT1G34310.1
	AtARF13	AT1G34170.3
	AtARF14	AT1G35540.1
	AtARF15	AT1G35520.1
	AtARF16	AT4G30080.1
	AtARF17	AT1G77850.1
	AtARF18	AT3G61830.1
	AtARF19	AT1G19220.1
	AtARF20	AT1G35240.1
	AtARF21	AT1G34410.1
	AtARF22	AT1G34390.1
	AtARF23	AT1G43950.1

The information of *Arabidopsis ARF* genes was from http://www.arabidopsis.org/.

Table S2 The qRT-PCR primer sequences of DnARF family genes.

	Up	Down
DnARF2a	GTCACCTCCAGCAAAGCC	CCCTCAGAAAAATAAAAGC
DnARF7	GTGGAGTGTGTTTGTAAG	CCAAGGGACACTTGCATG
DnARF6	CAGATGACATTGCAGCCT	TGCCTCTTGGGTTGTCCTC
DnARF11	CCAGAGGCAGATCAAAACG	CAAAGGTACTCCATCCTGTC
DnARF1	GAAACTGATCAAAGTGAAG	AACAAATATGCTCCAACC
DnARF3	CCAGCTTCGAGCAGAGGC	GCGCCACTCTAGGCCGTG
DnARF16a	GAACTTACCTTAGGAAGC	ACACAGAGATCACCATTC
DnARF2b	GCTATTACCACTGGAACC	AAGGGGAAACTCTCTCAG
DnARF19a	CTCTACATGCGGATACAG	GTCTCTTGCCTGCAGTTC
DnARF4	GTGACTGATGTGGTCTAC	CAACAAGTTTCTTCTTATGG
DnARF16b	CACGCCCAAAGTCCTGTGG	CTTGGAAATATAGTCTCTGC
DnARF10	CAACCGGTCAGAGATAGAG	CACAACTGAATCACCAGC
DnARF19b	CTTCAGCCAGTTAATTCTG	CAAACAAGCTCCAACCAGTC
DnARF17	CTTCTTTCAATGCAATGTC	GAGGACATAGATGGTTTG
